# An Ideal Amalgam Filling

**Published:** 1904-06

**Authors:** E. R. Batty

**Affiliations:** Alma, Neb.


### AN IDEAL AMALGAM FILLING.

By E. R. Batty, Alma, Neb.

Adjust the rubber dam to the tooth which is to be operated upon. Open into the cavity and remove all decay, care being taken not to cut away any more enamel than is necessary to allow proper access and to prevent the recurrence of decay. Prepare the margins in a symmetrical form and uolish as smooth as possible. Sterilize the cavity with a good germicide and dry well with hot air. Cleanse with chloroform to remove any oily or foreign sub stance and dry again with hot air. The cavity is now ready to be filled. Line with cement to the inside margin of the enamel and place a coating of amalgam over it before the cement has set. When the cement has become sufficiently hard proceed with the completion of the filling, using the amalgam with as little mercury as possible and malleting well. By this method we have a perfectly sealed cavity where decay will not recur. The dentine is protected from thermal changes and the walls will not turn dark. We also have better retention and a stronger tooth.—*Review.*

